# Construction of a five-gene prognostic model based on immune-related genes for the prediction of survival in pancreatic cancer

**DOI:** 10.1042/BSR20204301

**Published:** 2021-07-01

**Authors:** Bo Liu, Tingting Fu, Ping He, Chengyou Du, Ke Xu

**Affiliations:** 1 Department of Hepatobiliary Surgery, Pidu District People’s Hospital of Chengdu, Chengdu, Sichuan, China; 2 Department of Hepatobiliary Surgery, The Third Affiliated Hospital of Chengdu Medical College, Chengdu, Sichuan, China; 3 Department of Hepatobiliary Surgery, The First Affiliated Hospital of Chongqing Medical University, Chongqing, China; 4 Department of Nosocomial Infection Control, The Third Affiliated Hospital of Chengdu Medical College, Chengdu, Sichuan, China; 5 Department of Oncology, Clinical Medical College and The First Affiliated Hospital of Chengdu Medical College, Chengdu, Sichuan, China

**Keywords:** bioinformatics, immune-related genes, pancreatic cancer, prognostic model

## Abstract

**Purpose:** To identify differentially expressed immune-related genes (DEIRGs) and construct a model with survival-related DEIRGs for evaluating the prognosis of patients with pancreatic cancer (PC).

**Methods:** Six microarray gene expression datasets of PC from the Gene Expression Omnibus (GEO) and Immunology Database and Analysis Portal (ImmPort) were used to identify DEIRGs. RNA sequencing and clinical data from The Cancer Genome Atlas Program-Pancreatic Adenocarcinoma (TCGA-PAAD) database were used to establish the prognostic model. Univariate, least absolute shrinkage and selection operator (LASSO) and multivariate Cox regression analyses were applied to determine the final variables of the prognostic model. The median risk score was used as the cut-off value to classify samples into low- and high-risk groups. The prognostic model was further validated using an internal validation set of TCGA and an external validation set of GSE62452.

**Results:** In total, 142 DEIRGs were identified from six GEO datasets, 47 were survival-related DEIRGs. A prognostic model comprising five genes (i.e., *ERAP2, CXCL9, AREG, DKK1*, and *IL20RB*) was established. High-risk patients had poor survival compared with low-risk patients. The 1-, 2-, 3-year area under the receiver operating characteristic (ROC) curve of the model reached 0.85, 0.87, and 0.93, respectively. Additionally, the prognostic model reflected the infiltration of neutrophils and dendritic cells. The expression of most characteristic immune checkpoints was significantly higher in the high-risk group versus the low-risk group.

**Conclusions:** The five-gene prognostic model showed reliably predictive accuracy. This model may provide useful information for immunotherapy and facilitate personalized monitoring for patients with PC.

## Introduction

Pancreatic cancer (PC) is a highly malignant cancer and the seventh leading cause of mortality worldwide [1]. It is predicted that PC will become the second leading cause of cancer-related deaths by the year 2030 in U.S.A. [2]. Thus far, surgical therapy is the only curative strategy for resectable PC. However, only 10% of the patients are able to undergo standard resection at diagnosis due to the presence of atypical symptoms and the lack of effective imaging examination and diagnostic biomarkers in the early stage of disease [3]. At the time of diagnosis, most patients present with unresectable disease, characterized by nodal metastases, vascular invasion, or distant metastases [4]. Nevertheless, even patients who undergo surgical resection may not achieve satisfactory survival. Therefore, early detection and development of novel therapeutic strategies are urgently warranted to improve the survival of patients with PC.

The immune system plays a pivotal role in tumorigenesis and the progression of human malignancy [5]. A growing body of evidence suggests that the use of immunotherapy could result in favorable outcomes in cancer therapy. Blockade of immune checkpoints has shown substantial survival benefit for patients with several types of cancer, such as hepatocellular carcinoma, renal cell carcinoma, and melanoma [6–8]. Tumor cells could escape recognition and elimination by the immune system, induce immune tolerance, and promote their own growth and metastasis by secretion of immunosuppressive cytokines and regulation of the expression of immunoregulatory molecules [9,10]. Previous studies highlighted that the immune-related genes (IRGs) were associated with the prognosis of several types of cancer [11–13]. However, few studies investigated the role of IRGs in PC. Hence, the identification of genes with prognostic potential and construction of an effective predictive model may be useful for individualized management and assessment of prognosis in patients with PC.

In the present study, we utilized the Robust Rank Aggregation (RRA) method to identify differentially expressed genes (DEGs) between pancreatic tumors and adjacent normal tissues using six microarray datasets obtained from the Gene Expression Omnibus (GEO). Subsequently, univariate Cox regression was employed to identify survival-related differentially expressed immune-related genes (DEIRGs). Furthermore, least absolute shrinkage and selection operator (LASSO) Cox regression and multivariate Cox regression analyses were utilized to construct a prognostic model comprising survival-related DEIRGs. The median risk score calculated by the model was used to classify patients into high- and low-risk groups. The association between the model and immune cell infiltration was investigated. In addition, the expression of immune checkpoints in the low- and high-risk groups was compared. The aimed of the present study was to identify the survival-related biomarkers and therapeutic targets, establish a predictive model, and provide a basis for immunotherapy in patients with PC.

## Materials and methods

### Gene expression datasets

Six gene expression datasets (i.e., GSE15471, GSE60979, GSE62165, GSE71989, GSE91035, GSE102238) of PC were obtained from the GEO. All datasets met the following criteria: (1) included tumor and adjacent tissues of human PC; (2) comprised case and control groups; (3) contained >20 samples. Detailed information regarding these datasets is listed in Supplementary Table S1. A total of 170 PC sample profiles with available survival data were generated from The Cancer Genome Atlas-Pancreatic Adenocarcinoma (TCGA-PAAD) dataset. A training dataset with 102 samples and an internal validation dataset with 68 samples were randomly generated from the TCGA-PAAD dataset in a ratio of 3:2. In addition, a microarray dataset (GSE62452) containing 64 samples with survival data was obtained from the GEO for external validation. The characteristics of the training and the validation datasets are listed in Table 1.

**Table 1:** The clinicopathological characteristics of patients in training set and internal validation set of TCGA and an external GEO validation set

Characteristics	TCGA training set	TCGA validation set	GSE62452 validation set
	*n*=102	*n*=68	*n*=64
**Age at diagnosis (years)**	64.23 ± 10.68	64.79 ± 11.11	NA
**Gender (%)**			NA
Female	53 (52.0)	25 (36.8)	
Male	49 (48.0)	43 (63.2)	
**Tumor stage (%)**			NA
T1+T2	17 (16.7)	10 (14.7)	
T3+T4	83 (81.4)	58 (85.3)	
Not reported	2 (2.0)	0 (0.0)	
**Node stage (%)**			NA
N0	30 (29.4)	17 (25.0)	
N1	70 (68.6)	48 (70.6)	
Not reported	2 (2.0)	3 (4.4)	
**Pathologic stage (%)**			
I+II	95 (93.1)	65 (95.6)	47 (73.4)
III+IV	5 (4.9)	3 (4.4)	16 (25.0)
Not reported	2 (2.0)	0 (0.0)	1 (1.6)
**Histologic grade (%)**			
G1+G2	75 (73.5)	44 (64.7)	33 (51.6)
G3+G4	25 (24.5)	24 (35.3)	30 (46.9)
Not reported	2 (2.0)	0 (0.0)	1 (1.6)
**Events (%)**			
Alive	44 (43.1)	36 (52.9)	16 (25.0)
Dead	58 (56.9)	32 (47.1)	48 (75.0)
**Overall survival time (years)**	14.12 (9.21–22.02)	15.86 (11.48–22.84)	15.45 (9.20–27.63)

Abbreviation: NA, not available.

### Identification of DEIRGs

The *Limma* package was utilized to identify DEGs between tumor tissues and adjacent normal tissues of each dataset in the R platform (v3.6.1) [14]. The *RobustRankAggreg* package, which is based on the RRA method, was employed to normalize multiple datasets and conduct gene integration analysis for the identification of the most significant DEGs [15]. Genes with |log_2_ fold change| >1 and adjusted *P*-value <0.05 were selected as significant DEGs.

### Screening for survival-related DEIRGs

The IRG list (1811 genes) was obtained from the Immunology Database and Analysis Portal (ImmPort) [16]. DEIRGs were obtained by intersecting the IRG and DEG lists identified from the six GEO datasets. Subsequently, we performed univariate Cox regression analysis of DEIRGs to identify survival-related DEIRGs. Genes with *P*-value <0.01 were selected as survival-related DEIRGs.

### Functional enrichment analysis of DEIRGs

Gene Ontology (GO) enrichment analysis and Kyoto Encyclopedia of Genes and Genomes (KEGG) pathway analysis were carried out by the *Clusterprofiler* package to investigate the potential function of DEIRGs [17]. Adjusted *P*-value less than 0.05 was selected as the cut-off criteria for GO terms and KEGG pathway.

### Construction of a prognostic model

In the training set, LASSO regression through the *glmnet* package was utilized to determine the most powerful prognostic genes among the survival-related DEIRGs [18]. Next, multivariate Cox stepwise regression was applied to determine the best prognostic model. Subsequently, a prognostic model was established using a linear combination of the relative gene expression values (Exp_i_) and coefficient (β_i_) generated in the multivariate Cox regression. The risk score calculation formula is as follows: Risk score = Exp_1_ × β_1_+ Exp_2_ × β_2_ +……+ Exp_n_ × β_n_. The median risk score was used as the cut-off value to classify the PC samples into low- and high-risk groups. Kaplan–Meier curves analysis and log-rank test were performed to identify differences in survival. Time-dependent receiver operating characteristic (ROC) curve analysis was applied to evaluate the predictive ability of the prognostic model via the *timeROC* package [19]. Furthermore, we performed univariate and multivariate Cox regression analyses to investigate the independent factors between the risk score and clinical parameters, including age, sex, T stage, N stage, AJCC stage, and histologic grade. In addition, we performed gene set enrichment analysis (GSEA) utilizing the *ClusterProfiler* package to investigate the significantly enriched pathways between high- and low-risk groups in TCGA-PAAD dataset. We retrieved KEGG gene sets (c2.cp.kegg.v7.0.symbols.gmt) by using the *msigdbr* package.

### Correlation analysis of risk score and immune cells infiltration

Tumor Immune Estimation Resource (TIMER) is a comprehensive analytical web tool, which includes 10897 samples across 32 cancer types from TCGA to estimate the abundance of six tumor-infiltrating immune cell (TIIC) subsets (B cells, CD4 T cells, CD8 T cells, macrophages, neutrophils, and dendritic cells) [20]. Immune cells infiltration levels of PC patients which obtained from TIMER were applied for exploring the correlation between risk score and immune cells infiltration.

### Comparison of relative expression of immune checkpoints in low- and high-risk groups

Immunotherapy has achieved promising results in the treatment of many cancers in recent years. The most efficient strategy focused on the blockade of the immune checkpoints [21]. We compared the expression of most characteristic immune checkpoints, including programmed cell death 1 (PDCD1), PDCD1 ligand 1 (PDCD1-L1), cytotoxic T lymphocyte-associated protein 4 (CTLA4), CD80, CD86, V-domain immunoglobulin suppressor of T-cell activation (VISTA), T cell immunoglobulin mucin receptor 3 (TIM3), and T cell Ig and immunoreceptor tyrosine-based inhibition motif (ITIM) domain (TIGIT), between low- and high-risk groups aiming to provide information to optimize immunotherapeutic strategies for patients with PC.

### Statistical analysis

R software v3.6.1 (www.r-project.org) was used for statistical analyses in the present study. The *Limma* package was used to obtain DEGs. Kaplan–Meier curves analysis and log-rank test were performed using the *survival* package. Time-dependent ROC curve analysis was applied via the *timeROC* package. Univariate and multivariate Cox regression analyses were employed to determine independent factors for OS. Spearman correlation analysis was applied to investigate the correlation between risk score and immune cells infiltration. Difference of expression of immune checkpoints in low- and high-risk groups were compared using Wilcoxon’s test. Analysis results with *P*-value <0.05 indicated statistical significance.

## Results

### Identification of DEGs

**Figure 1:**
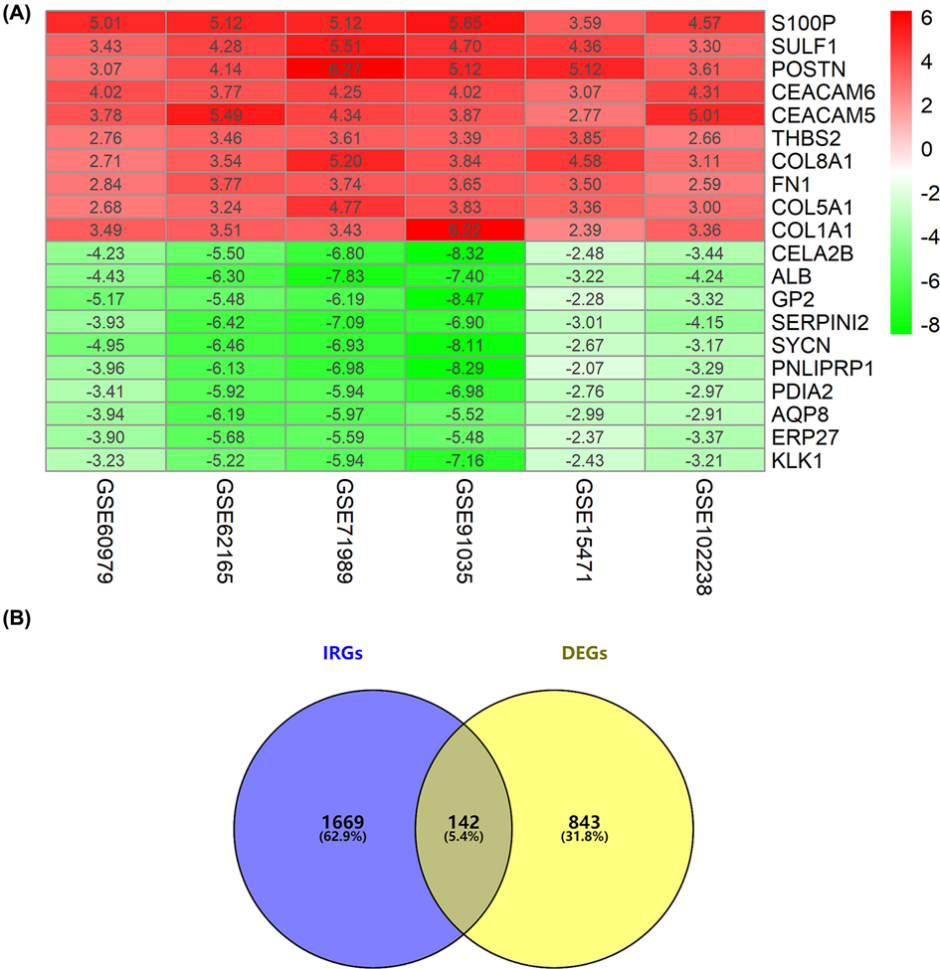
Identification of DEIRGs in GEO datasets (**A**) A heatmap of the top 20 up- and down-regulated DEGs in the integrated analysis. Red and green represent up- and down-regulated genes in each dataset, respectively. The numbers in each rectangle indicate the value of log_2_FC in each dataset calculated by ‘Limma’ package. The color gradient from green to red represents the log_2_FC from small to large. (**B**) Venn diagrams of the DEIRGs between DEGs in the integrated analysis and IRG list obtained from ImmPort.

A total of 985 DEGs, including 619 up- and 366 down-regulated genes were identified. The top 20 up- and down-regulated DEGs are shown in Figure 1A. We obtained a total of 142 DEIRGs by intersecting the DEG list with the IRG list (Figure 1B).

### GO and KEGG enrichment analysis for DEIRGs

**Figure 2:**
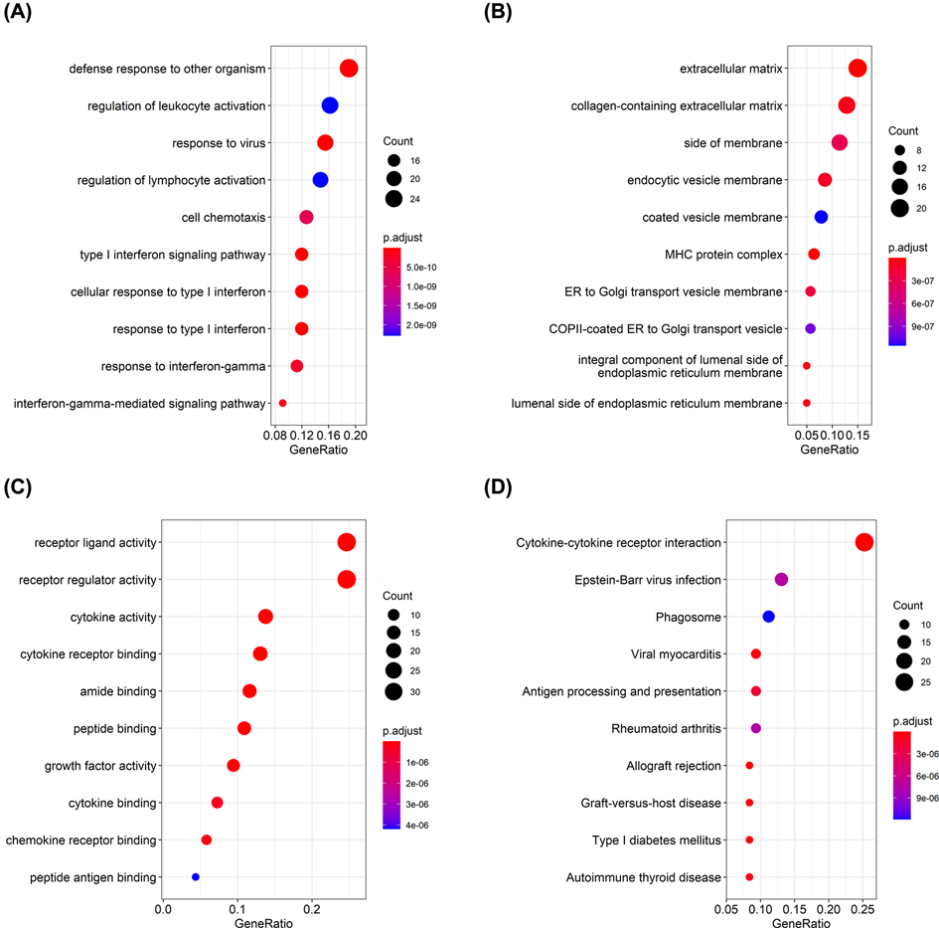
GO and KEGG analysis of DEIRGs (**A**–**C**) The top ten terms significantly enriched in the GO categories of BP, CC, and MF. (**D**) KEGG pathway analysis of DEIRGs. The bubble size is proportional to the number of DEIRGs involved. The color gradient from blue to red represents the *P*-value from small to large, respectively.

The following GO categories were enriched: biological process (BP), cellular component (CC), and molecular function (MF) (Figure 2A–C). The results showed that the significantly enriched terms were defense response to other organism (BP), extracellular matrix (CC), and receptor ligand activity (MF). Furthermore, according to the KEGG pathway analysis, the cytokine–cytokine receptor interaction pathway was markedly enriched (Figure 2D). The top ten pathways of DEIRGs are listed in Supplementary Table S2.

### Construction and internal validation of prognostic model

**Figure 3:**
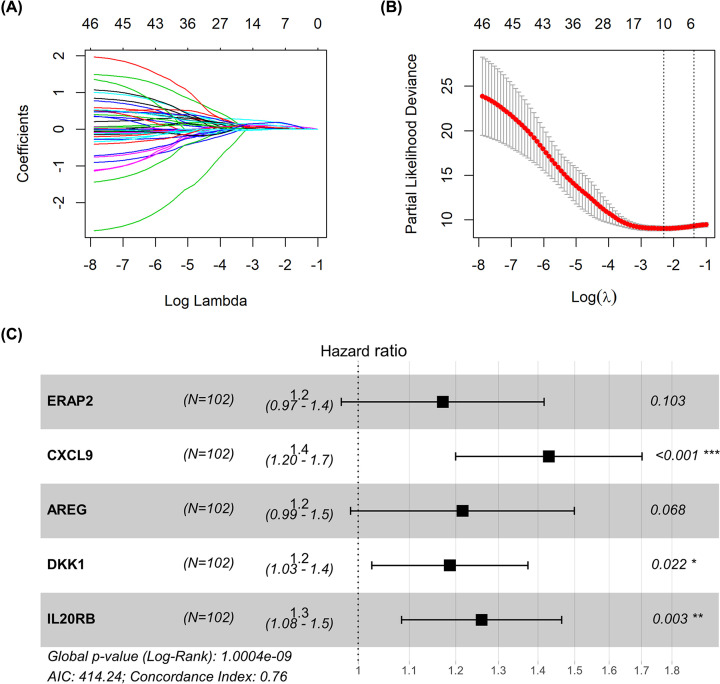
LASSO profiles and multivariate Cox regression analysis (**A**) LASSO coefficient profiles of 47 survival-related DEIRGs. (**B**) Ten-fold cross-validation result of the 47 survival-related DEGs. (**C**) Multivariate Cox regression analysis of the association between the five genes and overall survival in training set from TCGA.

We obtained 47 survival-related DEIRGs via univariate Cox regression analysis (Supplementary Table S3). LASSO Cox regression was applied to narrow down the number of relevant genes in the training dataset. The LASSO coefficient profiles of 47 survival-related DEIRGs are presented in Figure 3A. We obtained 12 genes with minimum partial likelihood deviance according to ten-fold cross-validation results (Figure 3B). Furthermore, the best prognostic model with the smallest Akaike Information Criterion was identified via multivariate Cox stepwise regression analysis. Finally, a prognostic model involving five genes, namely endoplasmic reticulum aminopeptidase 2 (ERAP2), amphiregulin (AREG), C–X–C motif chemokine ligand 9 (CXCL9), dickkopf-1 (DKK1), and interleukin-20 receptor subunit β (IL20RB) was constructed. Figure 3C shows that CXCL9, DKK1, and IL20RB exhibit the characteristics of independent prognostic factors in the training dataset. The prognostic risk score for each patient was calculated as follows: Risk score = (expression level of ERAP2 × 0.158) + (expression level of CXCL9 × 0.357) + (expression level of AREG × 0.195) + (expression level of DKK1 × 0.172) + (expression level of IL20RB × 0.231).

**Figure 4:**
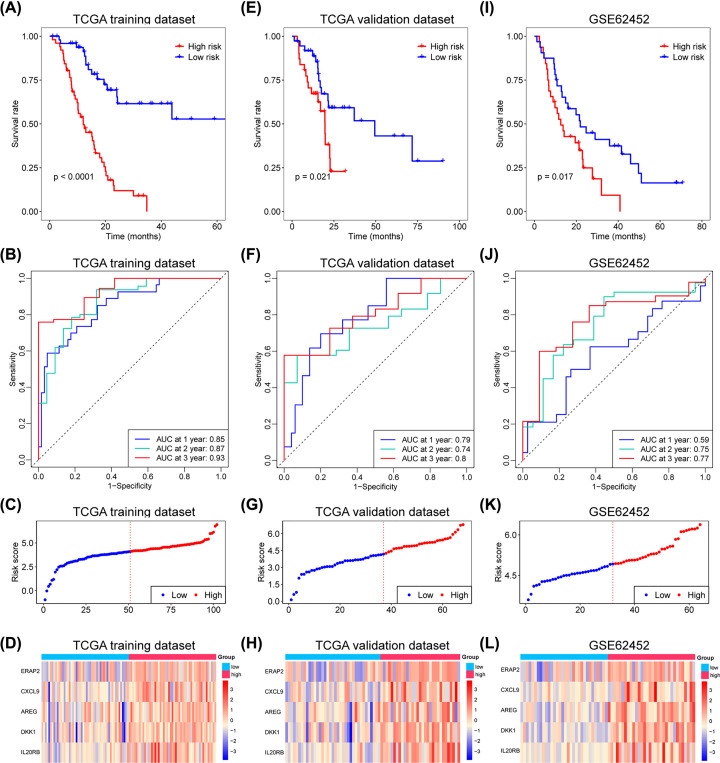
Establishment and validation of prognosis model Kaplan–Meier survival curves for low- and high-risk groups’ patients in TCGA training set (**A**), TCGA internal validation set (**E**) and external validation set (**I**). Time-dependent ROC curve analysis of the risk score model for predicting 1-, 2-, 3-year OS in TCGA training set (**B**), TCGA internal validation set (**F**) and external validation set (**J**). Distribution of the risk score (**C,G,K**). The expression of five survival-related IRGs in TCGA training set (**D**), TCGA internal validation set (**H**) and external validation set (**L**).

The patients in the training dataset were divided into low- and high-risk groups applying the median risk score as the cut-off criteria. The Kaplan–Meier curve shows that high-risk patients had a significant worse overall survival (OS) than low-risk group patients (Figure 4A). A time-dependent ROC curve was generated, and the area under the ROC curve (AUC) was calculated to assess the predictive ability of the model. In the training dataset, the 1-, 2-, and 3-year AUCs were 0.85, 0.87, and 0.93 (Figure 4B), respectively. The risk score distribution and the expression of the five genes in the training dataset are shown in Figure 4C,D. The prognostic model was further validated using the internal validation dataset. Similarly, patients with higher risk scores were associated with worse OS (Figure 4E). In the internal validation dataset, the 1-, 2-, and 3-year AUCs were 0.79, 0.74, and 0.8, respectively (Figure 4F). Figure 4G,H demonstrate the distribution of the risk score and a heatmap of the five gene expression data in the validation dataset, respectively. This indicates that this prognostic model is able to predict the OS of patients with PC in TCGA cohort.

### External validation of the prognostic model in GEO dataset

A GEO dataset (GSE62452) of PC with survival data was used as an external validation dataset to assess the predictive capability of the prognostic model. The risk score of each patient in the dataset was calculated using the formula of the model, and all patients were divided into high- and low-risk groups according to the median risk score. The OS observed in the high-risk group patients was significantly worse than that recorded in the low-risk group. In the external validation dataset, the 1-, 2-, and 3-year AUCs were 0.6, 0.75, and 0.77, respectively. External validation further confirmed the stable and accurate prognostic value of the present model in PC (Figure 4I–L).

### Evaluation of the independence of the prognostic model

**Figure 5:**
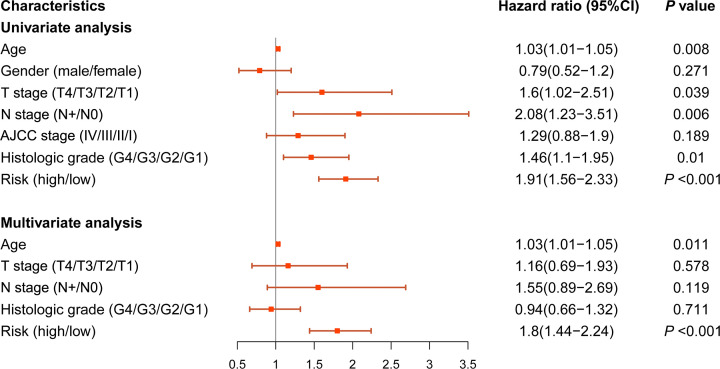
Univariate and multivariate Cox regression analyses for prognostic model and clinicopathological characteristics

To investigate the independent predictive ability of the prognostic model, we performed univariate and multivariate Cox regression analyses for the relationship between risk score and clinicopathological characteristics. The results showed that age, N stage, histologic grade, and risk score were associated with worse prognosis (Figure 5). Meanwhile, age and risk score were independent prognostic factors for OS. Moreover, higher risk scores were associated with advanced disease grade in TCGA-PAAD dataset (Supplementary Figure S1F).

### Correlation between the risk score and immune cell infiltration

**Figure 6:**
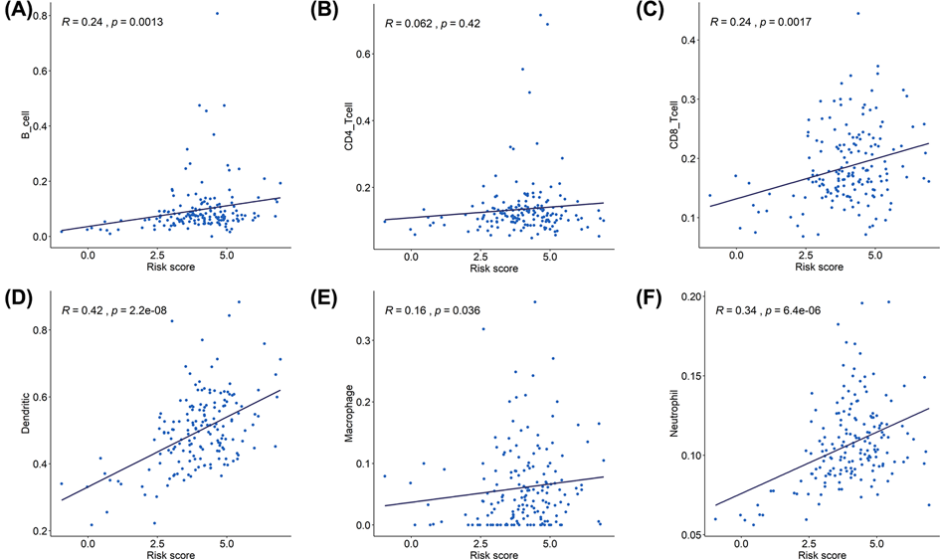
Association of risk score and immune cells infiltration (**A**) B cells, (**B**) CD4 T cells, (**C**) CD8 T cells, (**D**) dendritic cells, (**E**) macrophages, and (**F**) neutrophils.

We investigated the correlation between the risk score and the abundance of six tumor infiltrating immune cell subsets (i.e., B cells, CD4 T cells, CD8 T cells, macrophages, neutrophils, and dendritic cells). The results shown that the risk score was positively correlated with the infiltration of neutrophils and dendritic cells (Figure 6).

### Differences between the expression of immune checkpoints in the low- and high-risk groups

**Figure 7:**
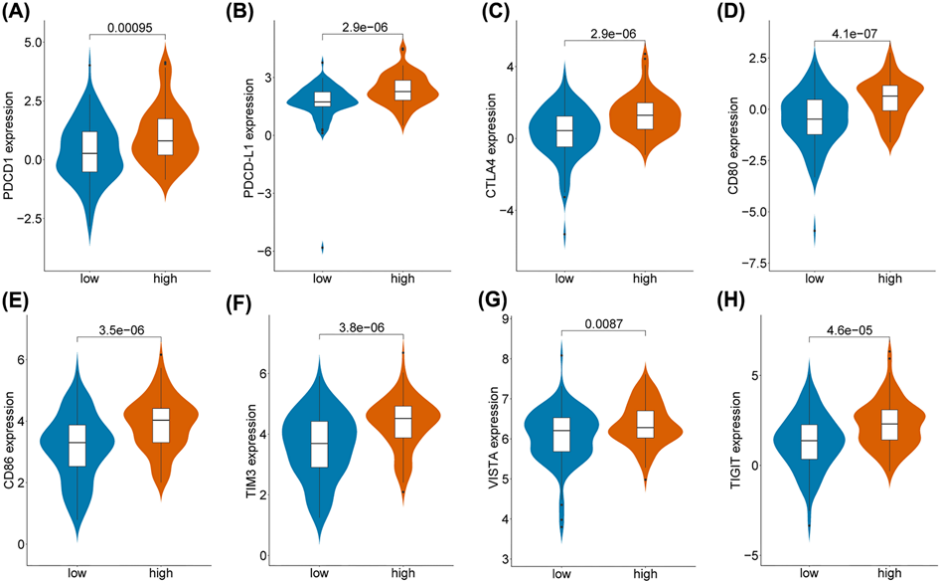
Different expression of immune checkpoints between low- and high-risk groups (**A**) PDCD1, (**B**) PDCD-L1, (**C**) CTLA4, (**D**) CD80, (**E**) CD86, (**F**) TIM3, (**G**) VISTA, (**H**) TIGIT.

We compared the relative expression of most characteristic immune checkpoints between the low- and high-risk groups. The expression of PDCD1, PDCD-L1, CTLA4, CD80, CD86, TIM3, VISTA, and TIGIT were significantly higher in the high-risk group compared with the low-risk group, indicating that immunosuppression may contribute to worse OS in high-risk patients (Figure 7).

### GSEA results

**Figure 8:**
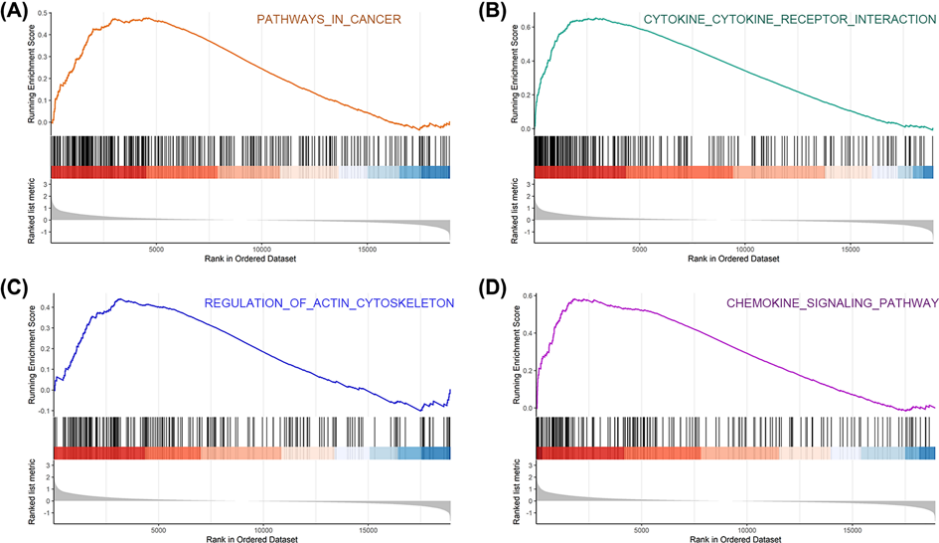
The top four significantly enriched pathways of this prognostic model (**A**) Pathways in cancer, (**B**) cytokine–cytokine receptor interaction, (**C**) regulation of actin cytoskeleton, (**D**) chemokine signaling pathway.

We performed GSEA to further investigate the different functional phenotype between the high- and low-risk groups. The results are listed in Supplementary Table S4. The four most significantly enriched pathways were: pathways in cancer, cytokine–cytokine receptor interaction, regulation of actin cytoskeleton, and chemokine signaling pathway (Figure 8).

## Discussion

PC remains a disease with dismal prognosis, namely poor survival and unfavorable therapeutic efficacy. Although numberous studies have investigated the relationship between IRGs and tumor prognosis, only a few focused on PC. In the present study, we identified DEIRGs with prognostic value in PC and established a prognostic model with five IRGs (i.e., ERAP2, CXCL9, AREG, DKK1, and IL20RB). Throughout the course of the study, 142 DEIRGs were identified by integrated analysis of six GEO datasets and intersected with an IRG list. The 47 survival-related DEIRGs were identified by univariate Cox regression. The five-IRG prognostic model was established by LASSO Cox regression and multivariate stepwise Cox regression. Notably, in the validation of the prognostic model, the 1-, 2-, and 3-year AUCs were 0.79, 0.74, and 0.8 (internal validation) and 0.59, 0.75, and 0.77 (external validation), respectively. The results indicated the reproducibility and generalizability of the prognostic model. Collectively, these results showed that this five-gene prognostic model was an effective predictive tool for OS.

Regarding the prognostic markers for PC, carbohydrate antigen 19-9 (CA19-9) is currently the most widely used marker in clinical practice. CA19-9 has shown a certain prognostic capability in both postoperative and advanced PC [22,23]. However, CA19-9 is not applicable to Lewis antigen-negative individuals, and false positive results may be obtained in cases with biliary infection and other malignant tumors [24,25]. Thus, there is a need to discover accurate prognostic markers for PC. In terms of immune-related molecules, a recent study reported that the expression of MET, 2′-5′-oligoadenylate synthetase 1 (OAS1) and 2′-5′-oligoadenylate synthetase like (OASL) was closely related to the progression of PC. Their expression was up-regulated in PC tissues and was associated with poor prognosis [26]. Similarly, from the perspective of RNA-binding proteins, a close relationship between OAS1 and the prognosis of PC was also found [27]. Guanylate-binding protein 2 (GBP2) is a guanylate-binding protein involved in response to viral or microbial infection; it is induced by Type I and Type II interferons. Recently, researchers found that GBP2 was highly expressed in PC and positively correlated with the expression of immune checkpoints (e.g., PDCD1, PDCD1-L1, CTLA4 etc.) Patients with PC and high expression of GBP2 were linked to a poor prognosis and an AUC of 0.69 for 3-year survival [28]. In addition, the effect of BRCA1/2 mutation on the prognosis of PC has been a focus of research in this field. Studies found that the BRCA1 rs1799966 polymorphism was correlated with the prognosis of PC. It was demonstrated that patients with advanced PC with BRCA2 mutation have a better response to platinum and a better prognosis [29–31]. Some non-coding RNAs and circulating tumor DNA have also been linked to the prognosis of PC; nevertheless, the usefulness of these markers is compromised by their limited predictive capability [32–35]. Therefore, the combination of these markers and construction of prognostic models may enhance the predictive capability.

Previously, researchers constructed some predictive models for PC based on prognosis-related genes. Representative studies were performed by Wu et al. [36] and Yan et al. [37] The former research group established a nine-gene signature to predict the OS of PC [36]. The latter research group constructed a four-gene prognostic model based on transcription factors and kinases associated with dysregulation genes [37]. However, these two prognostic models did not include immune-related DEG and did not investigate the association between the expression of immune checkpoints and risk score. In addition, the present model exhibited better capability in predicting the 1-, 2-, and 3-year survival of patients with PC.

Among the five genes of the model, ERAP2 is an aminopeptidase which is present in the lumen of the endoplasmic reticulum. It trims and generates peptide ligands for antigen presentation by major histocompatibility class I molecules [38,39]. Previous studies suggested that ERAP2 plays a pivotal role in vessel regeneration by inducing the migration and proliferation of endothelial cells [40,41]. ERAP2 could accelerate anti-tumor immune responses; hence, modulating the activity of ERAP2 may be a novel immunological strategy for cancer immunotherapy [42]. Chemokines are a family of small cytokines inducing directed chemotaxis, which can be found in most types of human cancer [43,44]. A member of the chemokine family, namely CXCL9, recruits leukocytes to sites of inflammation and plays a critical role in tumor progression. Previous studies reported that CXCL9 was correlated with worse OS in renal cell carcinoma, promoted tumor metastasis in melanoma, and enhanced the invasive ability of hepatocellular carcinoma [45–47]. Gao et al. demonstrated that CXCL9 was overexpressed in PC; this finding was consistent with the results of the present study [48]. They also found that CXCL9 could promote tumor progression in an orthotropic murine PAAD model by regulating the CD8^+^ T lymphocytes in the tumor microenvironment (TME). AREG is a ligand of epidermal growth factor receptor (EGFR), which is aberrantly expressed and plays a vital role in numerous types of cancer by mediating the motility, metastasis, and proliferation of cancer cells [49,50]. Stimulation of AREG increased the invasiveness, metastasis, and epithelial–mesenchymal transition of PC cells *in vivo* [51,52]. DKK1, a member of the DKK family, participates in the WNT/β-catenin pathway [53]. High expression of DKK1 was associated with aggressive features and shorter OS in patients with PC [54]. Previous studies demonstrated that DKK1 was correlated with accumulation of myeloid-derived suppressor cells in PC, contributing to the suppression of the responses of anti-tumor T cells [55]. DKK1 has been utilized as a potential target for immunotherapy in patients with myeloma [56]. IL20RB, a receptor of the IL20 subfamily, is involved in both amplified inflammatory and anti-inflammatory responses. Dysregulated expression of IL20RB has been observed in various studies, including the present study [57–59]. Overexpression of IL20RB was correlated with poor outcome in patients with papillary renal cell carcinoma, the ability of papillary renal cell carcinoma cells to invade and metastasize could be inhibited by silencing IL20RB in *vivo* [60].

Previous studies have shown that the TME of PC was infiltrated by immunosuppressive cells, but not effector lymphocytes [61,62]. Moreover, PC was characterized by a low proportion of tumor/stroma ratio in the tumor mass [63]. Notably, the stromal area of the TME was the main site of immune cell infiltration, which contributes to the poor outcome of PC [64]. In the present study, the correlation between the risk score and six subtypes of tumor-infiltrating immune cells was investigated. We observed that our prognostic model was positively associated with the infiltration of dendritic and neutrophil cells. According to the considerable research on immune checkpoints conducted in recent decades, immunotherapy has shown great curative potential for several types of cancer, such as hepatocellular carcinoma, melanoma, and bladder cancer [65–67]. Binding of PDCD1-L1 to its corresponding ligand PDCD1 negatively regulates the activity of immune cells and induces the immune evasion of tumor cells [68]. Previous studies indicated that PDCD1-L1 was overexpressed in patients with PC, and its down-regulation could inhibit the proliferation of pancreatic tumor cells [69]. CTLA4 is expressed by regulatory T cells, which are highly enriched in PC. The binding of CTLA4 to its ligands CD80 and CD86 leads to tumor cell immunosuppression. PC tumors are poorly immunogenic; hence, it is important to discover novel immune checkpoints for immunotherapy and develop more sophisticated treatment strategies. TIM3, belongs to the immunoglobulin superfamily and plays a dual role in regulating the immune response. It has been proved to be correlated with worse outcome in several types of cancer [70,71]. VISTA is a novel immune checkpoint overexpressed on CD68^+^ macrophages in PAAD. It is a potential immunotherapeutic target based on its high infiltration of the tumor environment and inhibition of T-cell activation [72]. TIGIT is expressed in several types of tumor cells and regulatory T cells; it is involved in immunosuppression and the immune evasion of cancer cells [73,74]. We compared the expression of immune checkpoints between patients in the low- and high-risk groups generated from our prognostic risk score model in TCGA-PAAD cohort. Interestingly, the expression levels of PDCD1, PDCD1-L1, CTLA4, CD80, TIM3, VISTA, and TIGIT in the high-risk group were notably higher than those measured in the low-risk group. These results indicated that the immunotherapeutic strategy of immune checkpoint blockade may be more effective for high-risk patients. GSEA revealed that the significantly enriched pathways in the high-risk group were associated with immune-related responses and tumorigenesis.

However, limitations in the present study should be realized. As our study was driven from statistical analysis of retrospective data, multicenter clinical trials and prospective research are required to further assess and validate this prognostic model. Meanwhile, additional experiment should be conducted to evaluate the expression of survival-related IRGs at protein level. Moreover, the biological function and mechanism of the IRGs in the prognostic model worth to be further elucidated in the future.

## Conclusion

In the present study, survival-related DEIRGs were identified, and a five-gene prognostic model with reliable predictive accuracy was constructed. The risk score calculated from the model is strongly correlated with immune cell infiltration in tumors and the expression of immune checkpoints. This model may provide new insight into the individualization of anti-tumor therapy and facilitate clinical monitoring of PC.

## Supplementary Material

Supplementary Figure S1 and Tables S1-S4

## Data Availability

The datasets generated and analyzed during the current study are available in TCGA official website repository (https://portal.gdc.cancer.gov/) and GEO (https://www.ncbi.nlm.nih.gov/geo/).

## Abbreviations

AREG: amphiregulin
AUC: area under the ROC curve
CA19-9: carbohydrate antigen 19-9
CC: cellular component
CTLA4: cytotoxic T lymphocyte-associated protein 4
CXCL9: C–X–C motif chemokine ligand 9
DEG: differentially expressed gene
DEIRG: differentially expressed immune-related gene
DKK1: Dickkopf-1
ERAP2: endoplasmic reticulum aminopeptidase 2
GBP2: guanylate-binding protein 2
GEO: Gene Expression Omnibus
GO: Gene Ontology
GSEA: gene set enrichment analysis
IL20RB: interleukin-20 receptor subunit β
IRG: immune-related gene
ITIM: immunoreceptor tyrosine-based inhibition motif
KEGG: Kyoto Encyclopedia of Genes and Genomes
LASSO: Least Absolute Shrinkage and Selection Operator
OAS1: 2′-5′-oligoadenylate synthetase 1
OS: overall survival
PAAD: pancreatic adenocarcinoma
PC: pancreatic cancer
PDCD1: programmed cell death 1
PDCD1-L1: PDCD1 ligand 1
ROC: receiver operating characteristic
RRA: Robust Rank Aggregation
TCGA: The Cancer Genome Atlas
TIGIT: T cell Ig and ITIM domain
TIM3: T cell immunoglobulin mucin receptor 3
TME: tumor microenvironment
VISTA: V-domain immunoglobulin suppressor of T-cell activation
