# Supplementary material for: Construction of a five-gene prognostic model based on immune-related genes for the prediction of survival in pancreatic cancer
Source: Biosci Rep. 2021 Jul 1;41(7):BSR20204301. doi: 10.1042/BSR20204301 (PMC8252190; doi:10.1042/BSR20204301)
Supplement: Supplementary Figure S1 and Tables S1-S4 [file BSR-2020-4301_supp.pdf]

Supplementary Materials

Supplemental Figure 1

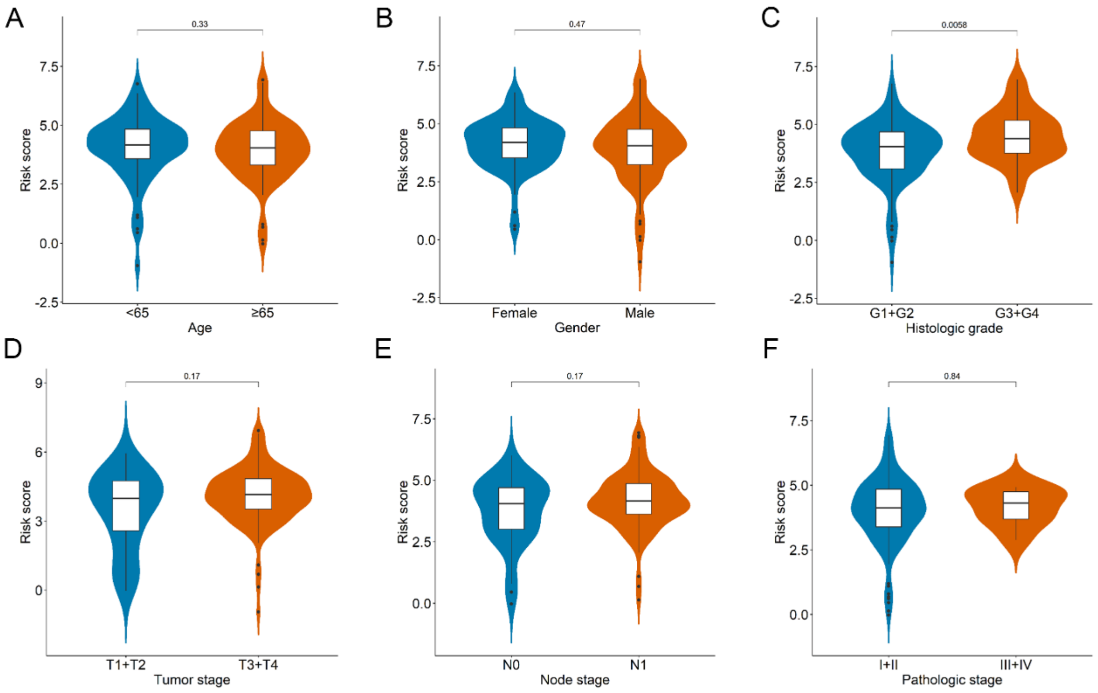

Supplemental Figure 1. Relationship between the risk score and clinicopathological characteristics: (A) age, (B) gender, (C) tumor stage, (D) node stage, (E) pathologic stage, and (F) histologic grade.

Supplemental Table 1. Characteristics of the datasets in this study.

Dataset	Normal	Tumor	Platform
GSE15471	39	39	GPL570
GSE60979	12	49	GPL14550
GSE62165	13	118	GPL13667
GSE71989	8	12	GPL570
GSE91035	8	25	GPL22763
GSE102238	50	50	GPL19072

Supplemental table 2. The result of KEGG pathway analysis of DEIRGs (ranked by *P* value).

ID	Description	Adjusted *P* value	Gene count	Gene symbol
hsa04060	Cytokine-cytokine receptor interaction	1.05E-13	27	CXCL14, CXCL9, CXCL5, CXCL12, CXCL3, CCL20, IL7R, TNFSF4, CCL18, CXCR4, EPO, GDF10, IL1RN, IL7, INHBA, LIF, TNFRSF11B, TNFSF13B, AMHR2, BMPR2, IL20RB, IL22RA1, IL4R, LIFR, TGFBR1, TNFRSF21, FAS
hsa05330	Allograft rejection	9.92E-08	9	HLA-A, HLA-B, HLA-C, HLA-DMB, HLA-DOA, HLA-DQA1, HLA-DQB1, HLA-F, FAS
hsa05332	Graft-versus-host disease	1.37E-07	9	HLA-A, HLA-B, HLA-C, HLA-DMB, HLA-DOA, HLA-DQA1, HLA-DQB1, HLA-F, FAS
hsa04940	Type I diabetes mellitus	1.62E-07	9	HLA-A, HLA-B, HLA-C, HLA-DMB, HLA-DOA, HLA-DQA1, HLA-DQB1, HLA-F, FAS
hsa05416	Viral myocarditis	1.78E-07	10	HLA-A, HLA-B, HLA-C, HLA-DMB, HLA-DOA, HLA-DQA1, HLA-DQB1, HLA-F, ICAM1, RAC2
hsa05320	Autoimmune thyroid disease	7.61E-07	9	HLA-A, HLA-B, HLA-C, HLA-DMB, HLA-DOA, HLA-DQA1, HLA-DQB1, HLA-F, FAS
hsa04612	Antigen processing and presentation	1.74E-06	10	CD74, CTSB, HLA-A, HLA-B, HLA-C, HLA-DMB, HLA-DOA, HLA-DQA1, HLA-DQB1, HLA-F
hsa05323	Rheumatoid arthritis	7.52E-06	10	HLA-DMB, HLA-DOA, HLA-DQA1, HLA-DQB1, ICAM1, CXCL5, CXCL12, CXCL3, CCL20, TNFSF13B
hsa05169	Epstein-Barr virus infection	7.52E-06	14	HLA-A, HLA-B, HLA-C, HLA-DMB, HLA-DOA, HLA-DQA1, HLA-DQB1, HLA-F, ICAM1, PSMD6, ISG15, IRF7, OAS1, FAS
hsa04061	Viral protein interaction with cytokine and cytokine receptor	1.19E-05	10	CXCL14, CXCL9, CXCL5, CXCL12, CXCL3, CCL20, CCL18, CXCR4, IL20RB, IL22RA1

Abbreviation: DEIRGs, differentially expressed immune-related genes. KEGG, Kyoto Encyclopedia of Genes and Genomes

Supplemental table 3. The result of Univariate Cox regression analysis for DEIRGs in TCGA PAAD dataset (*P* <0.01)

Gene symbol	HR	CI90	*P* value
GBP2	2.01	1.43-2.84	*P* < 0.001
PRDX1	1.76	1.2-2.58	0.004
TNFRSF21	1.64	1.2-2.24	0.002
FAS	1.54	1.15-2.07	0.004
S100A10	1.53	1.21-1.94	*P* < 0.001
S100A16	1.53	1.22-1.93	*P* < 0.001
TMSB10	1.49	1.14-1.95	0.004
OAS1	1.45	1.19-1.76	*P* < 0.001
PLAU	1.42	1.21-1.68	*P* < 0.001
SEMA3C	1.42	1.19-1.68	*P* < 0.001
PLAUR	1.4	1.14-1.73	0.002
NRP2	1.4	1.12-1.76	0.004
MICB	1.39	1.14-1.69	0.001
EDNRA	1.38	1.12-1.69	0.002
WNT5A	1.36	1.14-1.62	0.001
IL1RN	1.36	1.17-1.58	*P* < 0.001
OASL	1.34	1.16-1.54	*P* < 0.001
LTBP1	1.33	1.09-1.64	0.006
IFITM1	1.33	1.1-1.6	0.003
BST2	1.31	1.08-1.58	0.005
RSAD2	1.31	1.09-1.57	0.004
MX2	1.3	1.07-1.58	0.008
PPARG	1.3	1.11-1.51	0.001
AREG	1.3	1.14-1.48	*P* < 0.001
S100A6	1.29	1.08-1.55	0.005
IL20RB	1.28	1.15-1.42	*P* < 0.001
SDC1	1.28	1.08-1.52	0.005
NOD2	1.27	1.06-1.51	0.008
INHBA	1.27	1.09-1.47	0.002
NOX4	1.26	1.06-1.49	0.009
DKK1	1.25	1.13-1.37	*P* < 0.001
ERAP2	1.24	1.08-1.43	0.003
S100A14	1.23	1.09-1.38	0.001
IL22RA1	1.23	1.08-1.4	0.002
PTGS2	1.21	1.07-1.35	0.002
MMP12	1.18	1.06-1.32	0.002
S100P	1.18	1.08-1.3	*P* < 0.001
GREM1	1.18	1.06-1.32	0.003

CXCL9	1.17	1.07-1.29	0.001
S100A2	1.17	1.09-1.27	*P* < 0.001
PI3	1.15	1.06-1.26	0.001
CTSE	1.13	1.04-1.24	0.005
LCN2	1.13	1.04-1.24	0.006
CXCL5	1.12	1.04-1.19	0.001
CHGA	0.91	0.85-0.97	0.003
PAK3	0.85	0.76-0.95	0.006
NPPA	0.77	0.65-0.91	0.002

Abbreviation: DEIRGs, differentially expressed immune-related genes. TCGA, The Cancer Genome Atlas.

Supplemental table 4. The result of GSEA by comparing of high- and low- risk group in TCGA PAAD dataset (Top 10).

ID	SetSize	EnrichmentScore	NES	P values	P. adjust	Q values	Rank
PATHWAYS_IN_CANCER	325	0.47662	1.893014	0.001311	0.009578	0.006082	4515
CYTOKINE_CYTOKINE_RECEPTOR_INTERACTION	263	0.653825	2.562224	0.001348	0.009578	0.006082	2916
REGULATION_OF_ACTIN_CYTOSKELETON	212	0.440692	1.70072	0.001403	0.009578	0.006082	3188
CHEMOKINE_SIGNALING_PATHWAY	187	0.582715	2.220964	0.001427	0.009578	0.006082	1813
FOCAL_ADHESION	199	0.54239	2.073184	0.001431	0.009578	0.006082	3119
CELL_ADHESION_MOLECULES_CAMS	131	0.631299	2.305935	0.001508	0.009578	0.006082	2235
JAK_STAT_SIGNALING_PATHWAY	155	0.495857	1.839125	0.001508	0.009578	0.006082	3277
NATURAL_KILLER_CELL_MEDIATED_CYTOTOXICITY	131	0.614632	2.245055	0.001508	0.009578	0.006082	3240
T_CELL_RECEPTOR_SIGNALING_PATHWAY	108	0.535505	1.903617	0.001546	0.009578	0.006082	5143
LEUKOCYTE_TRANSENDOTHELIAL_MIGRATION	116	0.513062	1.839902	0.001553	0.009578	0.006082	3188

Abbreviation: GSEA, Gene set enrichment analysis. TCGA, The Cancer Genome Atlas. PAAD, pancreatic adenocarcinoma.
